# Lnc-CC3 increases metastasis in cervical cancer by increasing Slug expression

**DOI:** 10.18632/oncotarget.9519

**Published:** 2016-05-20

**Authors:** Binyuan Jiang, Ruili Sun, Shujuan Fang, Changfei Qin, Xi Pan, Li Peng, Yuehui Li, Guancheng Li

**Affiliations:** ^1^ Cancer Research Institute, Central South University, Changsha 410078, China; ^2^ Key Laboratory of Carcinogenesis, National Health and Family Planning Commission, Changsha 410078, China; ^3^ Key Laboratory of Carcinogenesis and Cancer Invasion, Ministry of Education, Changsha 410078, China

**Keywords:** cervical cancer, long noncoding RNA (LncRNA), lnc-CC3, tumor metastasis, Slug

## Abstract

Although screening has reduced mortality rates, metastasis still results in poor survival and prognosis in cervical cancer patients. We compared cervical cancer ESTs libraries with other ESTs libraries to identify candidate genes and cloned a novel cervical cancer-associated lncRNA, lnc-CC3. Overexpression of lnc-CC3 promoted migration and invasion by SiHa cervical cancer cells *in vitro* and *in vivo*, increased Slug expression, and reduced the expression of the epithelial cell marker E-cadherin. Conversely, lnc-CC3 knockdown altered SiHa cell morphology and increased the expression of E-cadherin, thereby suppressing migration and invasion. These results suggest lnc-CC3 may be a useful marker of metastasis in cervical cancer.

## INTRODUCTION

Cervical cancer is the second most common gynecological malignancy, and it remains a leading cause of cancer-related death among women in developing countries [1]. Although early screening reduces the mortality rates of cervical cancer patients, tumor metastasis negatively impacts survival even in early stage cervical cancer [2, 3]. Many studies have focused on cervical cancer in recent years, but the underlying molecular mechanisms remain unclear. The diversity of disease types and pathological characteristics complicates the study of cervical cancer molecular mechanisms [4, 5]; however, precision medicine may help to identify effective treatments on an individual basis in the future [6].

The accumulation of tumor-related genes is important in precision genetic diagnosis, which attempts to discover the functions of these genes to develop treatments and prognosis prediction strategies. For example, precision genetic diagnostics might help identify cervical patients for whom less radical surgeries that preserve future fertility are viable treatment options [7–9]. As more library construction and sequencing work is completed, the amount of genetic data available for tumor research continues to grow. Here, we used the National Cancer Institute's Cancer Genome Anatomy Project (CGAP) platform to explore genetic data related to cervical cancer. The CGAP Expressed Sequence Tags (ESTs) database currently contains 8,704,790 ESTs obtained from various normal and tumor tissues, and the number of ESTs continues to grow. The Relevant cDNA xProfiler tool compares gene expression between two specified library pools [10–11]; we compared cervical cancer ESTs libraries with other ESTs libraries in the CGAP database to identify novel cervical cancer-associated genes.

Less than 2% of the human genome codes for proteins, and large amounts of the genome are composed of noncoding transcripts [13]. For example, long noncoding RNAs (lncRNA), which are at least 200 bases in length and are involved in numerous physiological and pathological processes, are a large class of transcripts that yield little or no protein products. Thus far, only a small portion of lncRNAs have been characterized in detail, and all lncRNAs studied thus far regulate gene expression [14]. Some lncRNAs regulate vital cancer processes, including proliferation, apoptosis, metastasis, and drug resistance [15–18]. Many more lncRNAs that affect the expression of cancer-related genes will likely be identified in the future.

Epithelial-mesenchymal transition (EMT) is the process by which generally immotile epithelial cells convert to a motile mesenchymal phenotype. EMT contributes to tumor metastasis through the loss of cell-cell junctions [19]. E-cadherin is a single-span transmembrane protein primarily located within adherent junctions that plays an important role in EMT [20]. The E-cadherin/β-catenin complex forms intercellular junctions which maintain cell adhesion [21]. Downregulation of E-cadherin is an important characteristic of pathological cancer processes, such as tumor metastasis, and correlates with poor prognosis in numerous human tumors [22–24].

In this study, we cloned a novel lncRNA, lnc-CC3, and submitted it to the GenBank database. The accession number for lnc-CC3 is KT832804. Lnc-CC3 over-expression enhanced metastatic ability in SiHa cells by increasing Slug expression, which inhibits E-cadherin [25]. Together, these results indicate that lnc-CC3 could be a promising molecular target for the development of diagnostic and therapeutic strategies in cancer treatment. Additionally, our method represents a quick and cost-effective way to identify lncRNAs that affect the expression of tumor related genes.

## RESULTS

### *In silico* and PCR screening identified an EST highly expressed in SiHa cervical cancer cells

**Figure 1:**
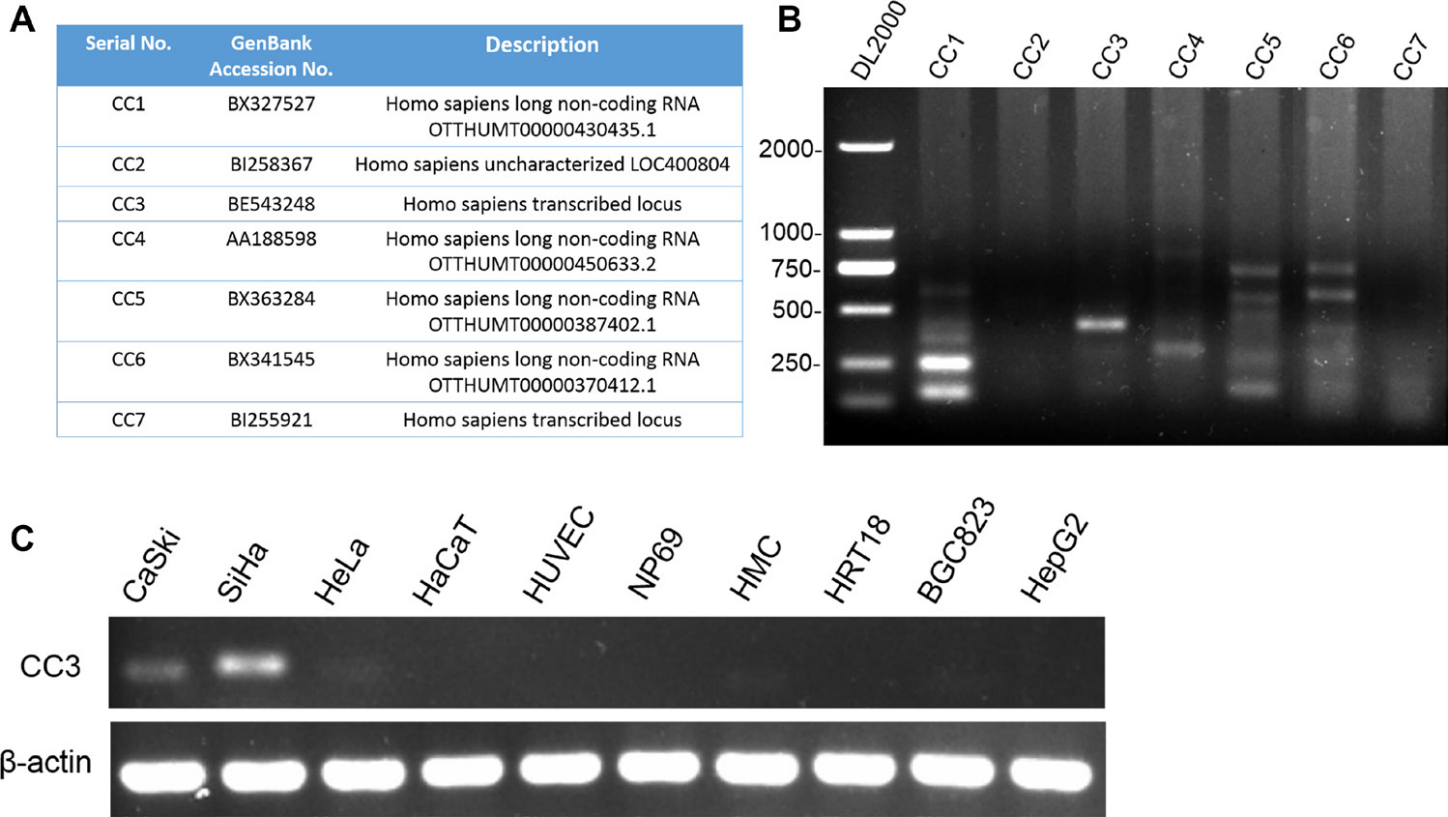
Screening for new genes associated with cervical cancer (**A**) Genbank ID and description of the 7 identified ESTs. (**B**) PCR showed that CC3 was present as a single band in the gel. (**C**) CC3 was expressed in cervical cancer cell lines; expression was higher in SiHa cells than in HeLa and CaSki cells.

After using cDNA X Profiler to compare the cervical cancer ESTs libraries with other ESTs libraries in the CGAP database, we filtered out known gene duplicates and intron-less ESTs and identified 7 ESTs for further study (Figure 1A). We evaluated the presence and transcript variety of these ESTs in CaSki cervical cancer cells using PCR; only CC3 formed a single band in the gel and met the RACE assay requirement (Figure 1B). CC3 expression was then tested in a panel of 10 cell lines; CC3 was expressed in all cervical cancer cell lines, and expression levels were higher in SiHa cells than in HeLa or CaSki cells. CC3 was not expressed in the normal HaCaT, HUVEC, and NP69 cell lines or in HRT18 and HepG2 cancer cells, and CC3 expression was very low in normal HMC cells and HeLa and BGC823 cancer cells (Figure 1C).

### CC3 is a novel lncRNA conserved in primates

**Figure 2:**
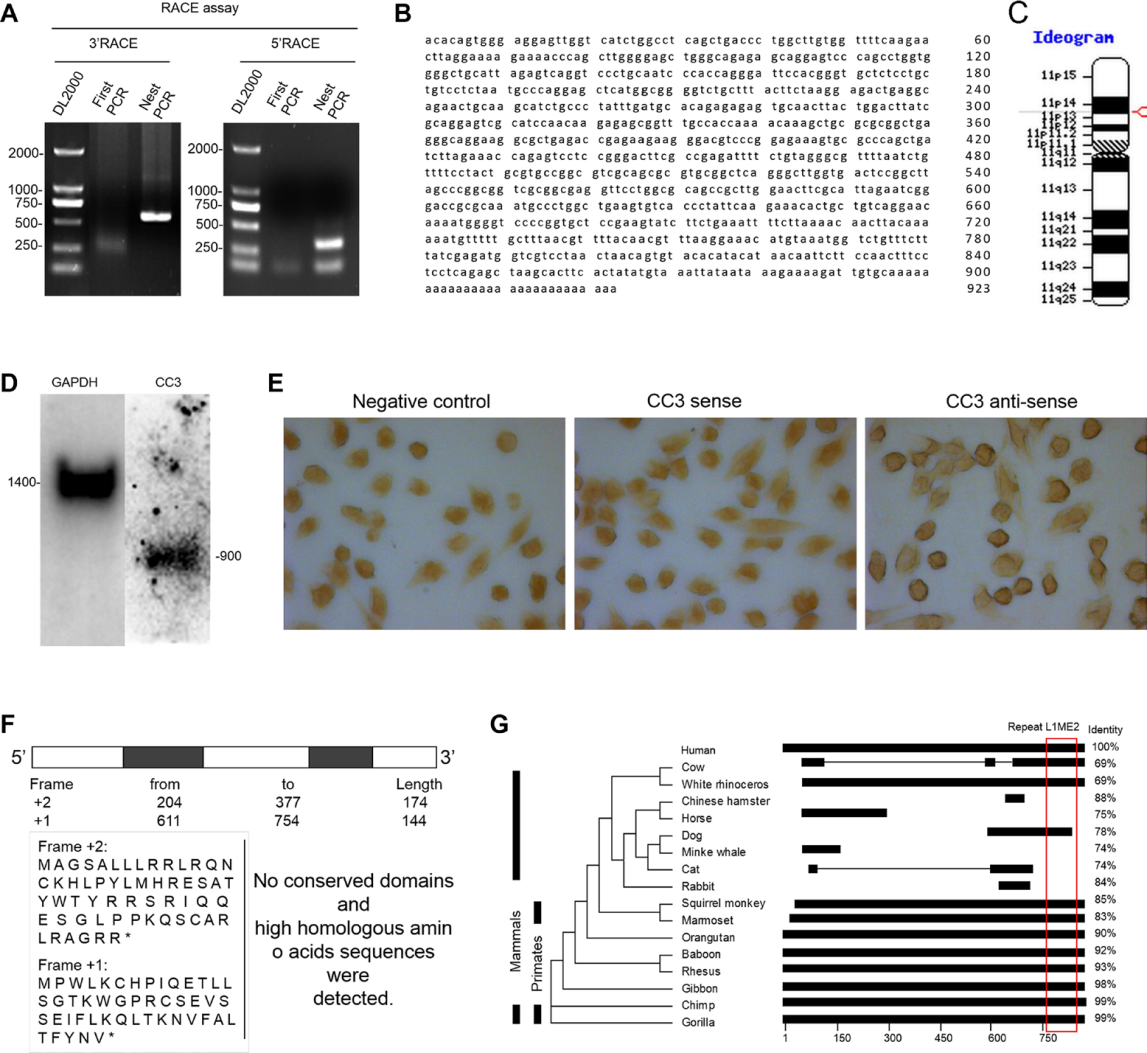
lnc-CC3 full length clone and sequence analysis (**A**) Electrophoresis of the RACE product. (**B**) The full sequence of lnc-CC3. (**C**) The chromosomal location of lnc-CC3 was determined using NCBI MapViewer; the red label indicates its position. (**D**) Northern blot results for lnc-CC3. (**E**) *In situ* hybridization of lnc-CC3 in SiHa cells; the photograph was taken at 200× magnification. (**F**) Putative proteins encoded by lnc-CC3 as predicted by ORF Finder; predicted proteins were subject to Blastp. (**G**) Conservation analysis of lnc-CC3 was conducted using Clustal Omega; repeat sequence analysis was conducted using RepeatMasker.

The 3′-RACE and 5′-RACE products were sequenced to determine the full sequence of CC3 (Figure 2A). The 923bp nucleotide sequence (Figure 2B) was located on chromosome 11 and contained two exons and poly-a tail (Figure 2C); the length of lnc-CC3 RNA was confirmed by northern blot assay (Figure 2D). *In situ* hybridization indicated that CC3 was mainly located in the cytoplasm of SiHa cells (Figure 2E). ORF Finder was used to analyze the protein coding potential of CC3; no valid Kozak sequence was found in the ORFs, and the predicted amino acid sequence of the longest ORF block (174bp) had no homologous protein or conserved domain (Figure 2F). We defined CC3 as an lncRNA based on standard features [26, 27]. UCSC Genome Browser and Clustal Omega were used to compare the genome assemblies of lnc-CC3 to other species, and conservation analysis indicated that lnc-CC3 is conserved in primates. RepeatMasker analysis identified a repeat L1ME2 element located between bp 761–887 in the lnc-CC3 sequence (Figure 2G); this region was also conserved in dog and cow and might help identify the function of lnc-CC3.

### Increased Lnc-CC3 expression is associated with stage III cervical cancer

**Figure 3:**
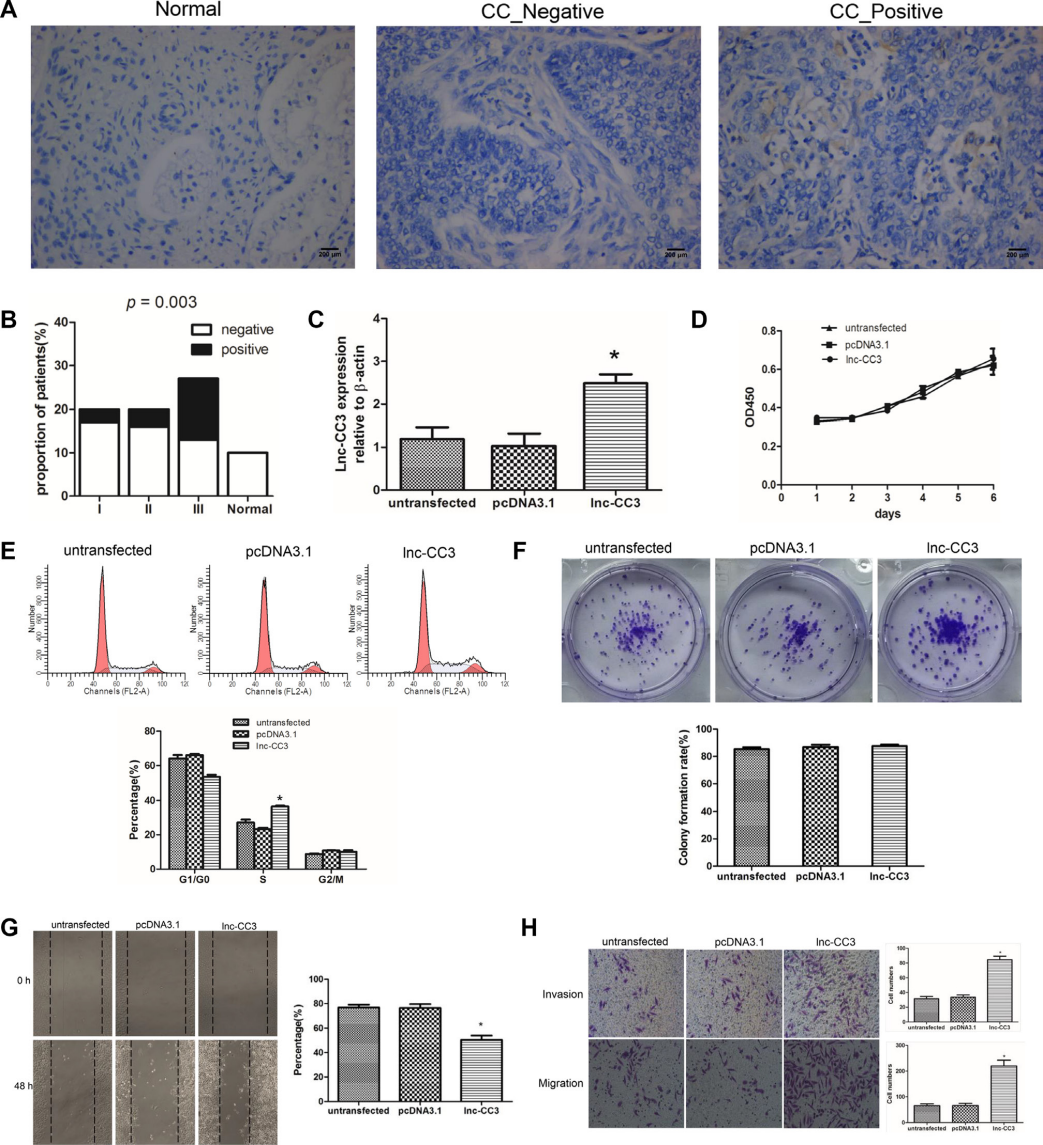
Lnc-CC3 over-expression increased migration and invasion in SiHa cells *in vitro* (**A**) *In situ* hybridization results for the cervical tissue microarray revealed that lnc-CC3 is mainly localized in the cytoplasm. Normal, normal cervical tissue; CC _ Negative, cervical cancer negative; CC _ Positive, cervical cancer positive. 200× magnification, scale bar = 200 μm. (**B**) Lnc-CC3 levels were elevated more frequently in stage III cervical cancer. Normal, normal cervical tissue; I, II, III represent the different cervical cancer stages. Statistical significance was determined using the χ^2^ test, *p* = 0.003. (**C**) Fold change in lnc-CC3 expression in SiHa cells was analyzed by qRT-PCR (*n* = 3); β-actin was used as an internal control. Data were analyzed using the 2^−ΔΔCT^ method. (**D**) Cell proliferation curve for SiHa cells determined using the CCK-8 assay (*n* = 3). (**E**) Cell cycle distribution of SiHa cells was determined by flow cytometry (*n* = 3). (**F**) Colony formation assay results for SiHa cells (*n* = 3). (**G**) Cell migration capacity was determined using a wound healing assay (*n* = 3). Photographs were taken immediately at 0 h and 48 h after wounding at 100× magnification at the same location in each well. (**H**) Representative images of the migration and invasion transwell assay; invaded cell number was determined by photograph at 200× magnification in five random views per chamber. Untransfected and pcDNA3.1 (+) plasmid transfected SiHa cell lines were used as controls; lnc-CC3 indicates lnc-CC3 overexpressing SiHa cells. Data are expressed as mean ± SD of independent experiments, *p < 0.05.

To further investigate the association between lnc-CC3 and cervical cancer, we examined lnc-CC3 expression in a tissue microarray containing specimens from 10 normal cervical tissues and 70 cervical cancer tissues (22 stage I, 20 stage II, and 28 stage III) using *in situ* hybridization. As in SiHa cells, lnc-CC3 was mainly localized in the cytoplasm of epithelial cells (Figure 3A). Lnc-CC3 expression was detected in 3/20 (15%) stage I, 4/20 (20%) stage II, and 14/27 (51.8%) stage III cervical tumor tissue specimens. Lnc-CC3 expression in cervical cancer did not differ depending on patient age (< 55 years or ≥ 55 years, *P* = 0.537), but was higher in stage III specimens compared to the other stages (*p* = 0.003) (Figure 3B, Tables S2–S3).

### Lnc-CC3 overexpression enhances migration and invasion in cervical cancer cells *in vitro*

Full length lnc-CC3 was recombined with eukaryotic expression plasmid pcDNA3.1 (+) and transfected into SiHa cells with Lipofectamine 2000. Cells stably overexpressing lnc-CC3 were harvested after G418-resistant filtration (Figure 3C). Cell proliferation curves indicated that lnc-CC3 did not promote SiHa cell proliferation (Figure 3D), and flow cytometry indicated that lnc-CC3 arrested SiHa cells in S phase (Figure 3E). Lnc-CC3 overexpression did not affect colony formation efficiency in the colony formation assay, but lnc-CC3 overexpressing SiHa colonies were larger than controls (Figures 3F, Figure S1). These data indicated that, although lnc-CC3 did not impact proliferation in SiHa cells, it did affect cell mobility. Wound healing and transwell assays showed that lnc-CC3 clearly increased the migration and invasion capacity of SiHa cells *in vitro* (Figure 3G, 3H).

### Lnc-CC3 knockdown altered SiHa cell morphology and suppressed migration and invasion *in vitro*

**Figure 4:**
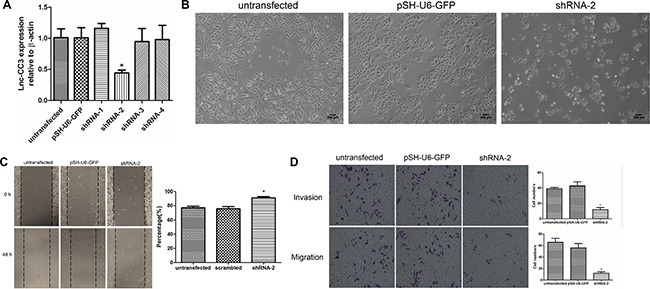
Lnc-CC3 knockdown suppressed migration and invasion in SiHa cells *in vitro* (**A**) Fold change in lnc-CC3 expression in SiHa cells was analyzed by qRT-PCR (*n* = 3); β-actin was used as an internal control. Data were analyzed using the 2^−ΔΔCT^ method. (**B**) The shape of lnc-CC3 knockdown SiHa cells changed from elongated to round. Photograph at 100× magnification, scale bar = 200 μm. (**C**) Cell migration capacity was determined by wound healing assay (*n* = 3). Photographs were taken immediately at 0 h and 48 h after wounding at 100× magnification, at the same location in each well. (**D**) Representative images of the migration and invasion transwell assay; invaded cell number was determined by photograph at 200× magnification in five random views per chamber. Data are expressed as mean ± SD of independent experiments, *p < 0.05.

To confirm the function of lnc-CC3 in cell migration and invasion, we established lnc-CC3 knockdown SiHa cells using shRNA. Four shRNA plasmid vectors and control vector were transfected into SiHa cells with Lipofectamine 2000, and stably transfected cells were harvested after puromycin-resistant filtration (Figure 4A). shRNA-2 reduced lnc-CC3 expression in SiHa cells, and the shape of these SiHa cell changed from elongated to round (Figure 4B). Wound healing and transwell assays indicated that lnc-CC3 knockdown reduced the metastasis and invasion capacity of SiHa cells (Figure 4C, 4D). Together, these results reconfirmed that lnc-CC3 affects SiHa cell migration and invasion *in vitro*.

### Lnc-CC3 increased the lung colonization capacity of SiHa cells

**Figure 5:**
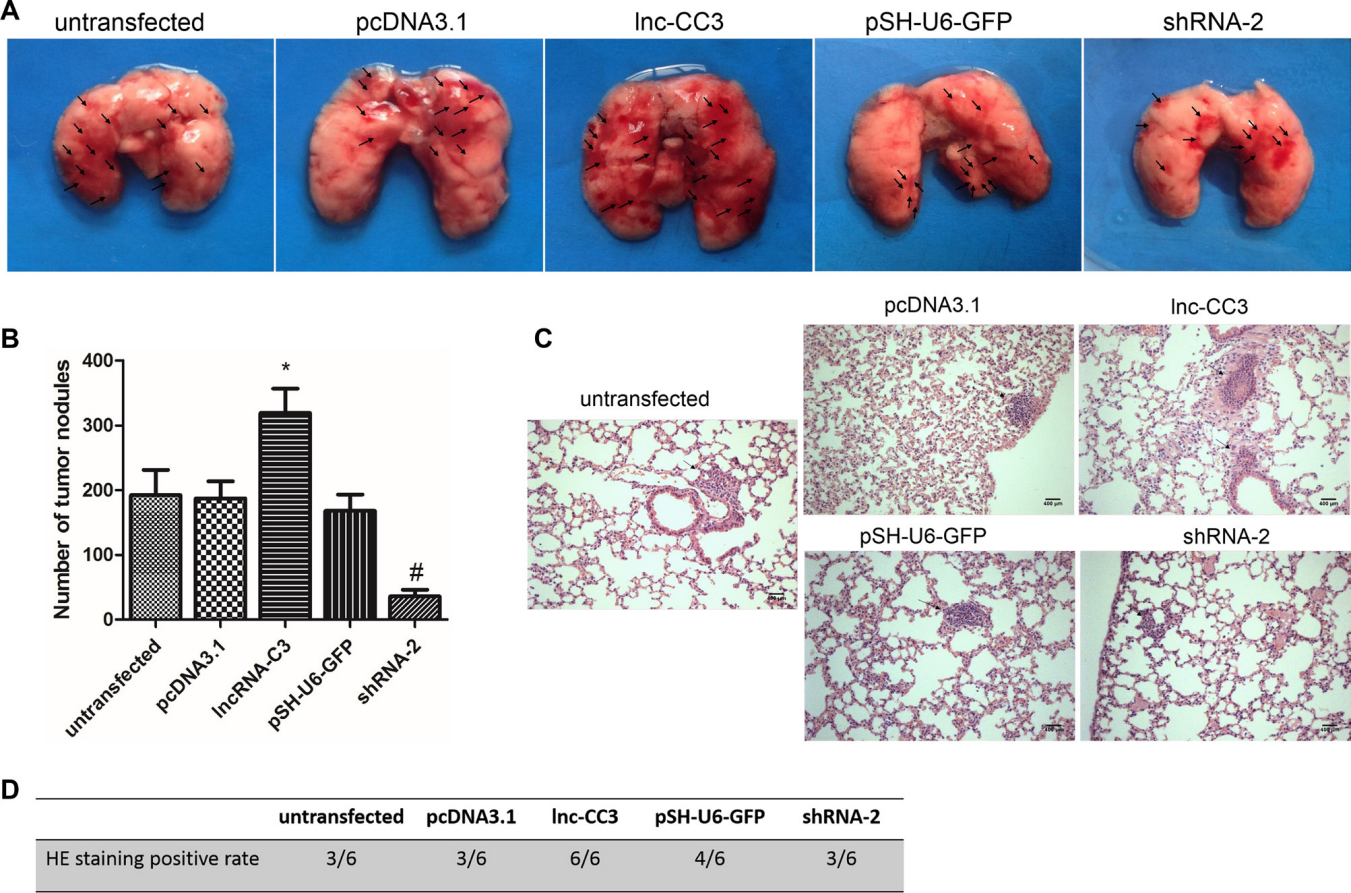
Lnc-CC3 enhanced the lung colonization capacity of SiHa cells (**A**) Representative images of nude mouse lungs; arrows indicate clusters of nodules on the lung surface. (**B**) Metastatic tumor nodules on the lung surface were counted under stereomicroscope. Each group contained 6 nude mice. (**C**) Hematoxylin-eosin staining of lung tumor nodules. Photographed at 100× magnification, scale bar = 400 μm. (**D**) Neoplasms were found in all pathological sections from lnc-CC3 overexpressing SiHa cells. Data are expressed as mean ± SEM of independent experiments (*n* = 6), *p < 0.05, ^#^*p* < 0.05.

We determined the effects of lnc-CC3 on the formation of pulmonary metastases by injecting SiHa cells into female nude mice via the tail vein. After 8 weeks, mice were sacrificed and the lungs were dissected. Injections of lnc-CC3 overexpressing SiHa cells increased the number of irregular hemorrhage sites on the lung surface (Figure 5A). Conversely, injections of lnc-CC3 knockdown SiHa cells reduced the number of tumor pulmonary metastases (Figure 5B). Body weights did not differ among the groups. Histopathology also indicated that lnc-CC3 increased the metastatic capacity of SiHa cells (Figures 5C, 4D). Thus, injections of cells overexpressing lnc-CC3 increased, and injections of lnc-CC3 knockdown cells decreased, lung colonization capacity *in vivo*.

### Lnc-CC3 may promote EMT

**Figure 6:**
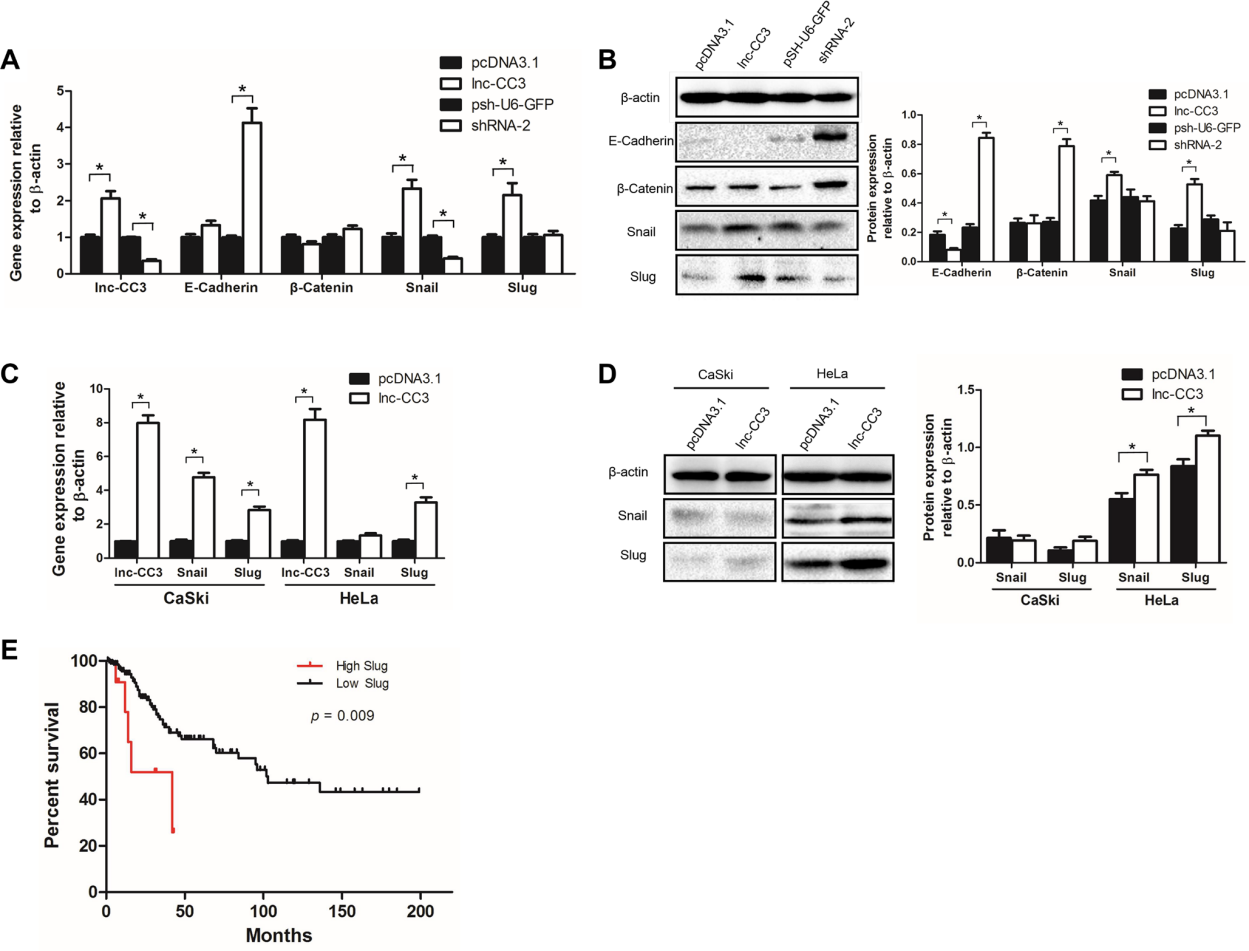
Lnc-CC3 promoted EMT in cervical cancer cells by increasing Slug expression (**A**) Lnc-CC3 overexpression increased the expression of Slug and Snail, and lnc-CC3 knockdown increased the expression of E-cadherin and β-catenin. (**B**) Changes in Slug, Snail, E-cadherin, and β-catenin protein levels in SiHa cells. The effects of lnc-CC3 overexpression on Snail and Slug mRNA (**C**) and protein (**D**) levels in CaSki and HeLa cells. The qRT-PCR data were analyzed using the 2^−ΔΔCT^ method and WB data were quantified by densitometry using ImageJ software. Data are expressed as mean ± SD of independent experiments (*n* = 3), *p < 0.05. (**E**) Overall survival using the Kaplan-Meier method in cervical cancer samples from TCGA with differing Slug expression; survival rates for cases with high (change fold ≥ 2) and low (change fold ≤ 2) Slug expression were compared, *p* = 0.009.

Epithelial-mesenchymal transition (EMT) is an essential process by which generally immotile epithelial cells adopt a motile mesenchymal phenotype. Malignant epithelial tumors display reduced intracellular adhesion and increased motility, which promotes the spread of cancer. Because *in vitro* and *in vivo* tests indicated that lnc-CC3 increased the migration and invasion capacity of SiHa cells, we investigated the effects of lnc-CC3 on EMT markers. Lnc-CC3 overexpression increased the expression of Slug and Snail (Figure 6A, 6B), which are transcriptional repressors of E-cadherin [28, 29], and reduced the expression of E-cadherin and degradation of the E-cadherin/β-catenin complex [25]; SiHa cells overexpressing lnc-CC3 thus displayed characteristics of mesenchymal cells. Conversely, lnc-CC3 knockdown increased the expression of E-cadherin and β-catenin, resulting in more adhesion in SiHa cells. Lnc-CC3 also increased Slug and Snail mRNA levels in CaSki and HeLa cells and Slug and Snail protein levels in HeLa cells (Figure 6C, 6D). In CaSki cells, Slug that is not transported into the nucleus as a transcription regulatory factor is rapidly degraded [30, 31]. This may explain the failure of lnc-CC3 overexpression to increase invasion and metastasis in CaSki cells (Figure S2). Furthermore, lnc-CC3 may increase invasion and metastasis in SiHa and HeLa cells specifically by increasing Slug protein and mRNA levels.

Because there was no record for lnc-CC3 in the TCGA (The Cancer Genome Atlas) database, we instead investigated Slug mRNA expression and its clinical significance in cervical cancer. Slug fold-change and clinical data for cervical cancer samples were downloaded from TCGA, and survival rates were compared in cases with high (change fold ≥ 2) and low Slug expression (change fold ≤ 2). The survival curve indicated that high Slug expression cases had a lower survival rate (Figure 6E); lnc-CC3 might therefore contribute to poor survival in cervical cancer by increasing Slug expression.

## DISCUSSION

In this report, we conducted a CGAP search to identify cervical cancer-related genes and, based on the results, cloned a novel primate-conserved long noncoding RNA, lnc-CC3. *In vitro* and *in vivo* tests indicated that lnc-CC3 increased metastasis in SiHa cervical cancer cells; lnc-CC3 may therefore be a useful diagnostic biomarker and therapeutic target in cervical cancer.

In recent years, researchers have developed platforms, including CGAP, TCGA, and ICGC, to share and mine genetic cancer data [32–35]. The NCI's Cancer Genome Anatomy Project focuses on transcription-related information, while TCGA and ICGC provide more comprehensive information, including genome, transcriptome, and epigenetic data as well as patient medical records. These platforms allow researchers to identify genetic mechanisms that might contribute to cancer “*in silico*” and then investigate these mechanisms in more detail with *in vivo* and *in vitro* experiments [36, 37].

In this study, we used the cDNA xProfiler tool to identify 100 unique cervical cancer ESTs from the cDNA library, but most of these ESTs were known gene duplicates or genome fragments; BLAST validation indicated that 7 of the ESTs were worthy of further study. Furthermore, only one EST could be evaluated using the RACE assay, so we cloned its full-length cDNA. Notably, 6 of 7 ESTs identified were uncharacterized lncRNAs, suggesting that almost all protein-coding genes relevant in cervical cancer have been cloned and added to the UniGene database. However, many recent studies have examined the roles of various lncRNAs in cancer; examples include MALAT1 in small cell lung carcinoma [38, 39], lncRNA-ATB in hepatocellular carcinoma, and NKILA in breast cancer [40, 41]. Many lncRNAs also participate in the progression of cervical cancer, affecting proliferation, apoptosis, metastasis, radio-resistance, and HPV [42–44]. Many potentially cancer-relevant lncRNAs have not yet been characterized, and future lncRNA studies may help greatly improve individualized diagnosis techniques [45, 46].

Here, we found that lnc-CC3 promoted SiHa cell metastasis by increasing Slug expression. Slug is a member of the SNAIL family of transcriptional repressors, which have been implicated in a variety of developmental and cellular processes, including cell motility and EMT induction [47, 48]. The loss of E-cadherin is a hallmark of EMT, and both SNAI1 (Snail) and SNAI2 (Slug) target E-cadherin. Snail and Slug bind to the E2-boxes in the proximal E-cadherin promoter and recruit different co-repressor complexes [49, 50]. In this study, lnc-CC3 overexpression increased Slug mRNA levels in SiHa, CaSki, and HeLa cells. Our results suggest that lnc-CC3 activated Slug expression, and future studies should investigate the mechanisms underlying this lnc-CC3/Slug/E-cadherin cascade in cervical cancer.

In conclusion, we cloned a novel cervical cancer-associated long noncoding RNA, lnc-CC3, based on information from bioinformatics databases. Lnc-CC3 promoted migration and invasion in cervical cancer cells by increasing Slug expression. In contrast, lnc-CC3 knockdown altered SiHa cell morphology and suppressed their metastatic capacity *in vitro* and *in vivo*. Further investigation of lnc-CC3 may confirm its utility as a novel diagnosis biomarker and therapy target. The research strategy employed here may also aid in the discovery of additional novel tumor-related lncRNAs and help improve future cervical cancer treatments.

## MATERIALS AND METHODS

### The screening of cervical cancer-associated ESTs

The CGAP cDNA X Profiler (http://cgap.nci.nih.gov/Tissues/xProfiler) was used to search cervical cancer ESTs in human ESTs libraries. Microdissected tissues, cell lines, and flow sorted cell tissue preparations were selected. Pool A contained cervical cancer ESTs libraries and Pool B contained all other cancer and normal tissue ESTs libraries. NCBI BLAST (http://blast.ncbi.nlm.nih.gov/Blast.cgi) was used to filter out known genes and intron-less sequences. The NCBI UniGene and ESTs database was used to further aid in identification. We designed PCR-primers to test the transcript variety of these ESTs to insure suitability for the RACE assay (Table S1).

### Cell culture

The following human cell lines were used and maintained by our lab: SiHa, HeLa, and CaSki (cervical cancer), HaCaT (human epidermal cell), HUVEC (human umbilical vein endothelial cell), NP69 (human nasopharyngeal epithelial cell), HMC (human glomerular mesangial cell), HRT18 (colorectal Cancer), BGC823 (gastric cancer), and HepG2 (hepatocellular carcinoma). All cell lines were cultured in RPMI 1640 medium with 10% FBS (Gibco, Carlsbad, CA, USA), 100 units/mL penicillin, and 100 g/mL streptomycin (Sigma Chemicals, St Louis, MO, USA).

### RNA isolation and cDNA preparation

Total RNA was extracted from cell lines using TRIzol reagent (Invitrogen, Carlsbad, USA) according to the manufacturer's instructions. All RNA samples were tested for integrity and purity with an ultraviolet spectrophotometer (OD260/OD280) and electrophoresis. First-strand cDNAs were synthesized from 2 μg total RNA using the First-Strand cDNA Synthesis kit (Fermentas, Waltham, MA, USA).

### Semi-quantitative RT-PCR and quantitative real-time PCR

A semi-quantitative RT-PCR assay was used to test the presence of ESTs and CC3 expression in different cell lines; β-actin was used as an internal control. The cDNA product was amplified with 2× Dream Taq Green PCR Master Mix (Thermo Scientific, Waltham, MA, USA) in a final volume of 20 μL. Reaction products were separated on 1% agarose gels containing ethidium bromide. Quantitative real-time PCR was conducted using the SYBR Premix ExTaq II kit (Takara, Dalian, China) in the MiniOpticon Real-Time PCR Detection System (Bio-Rad, Hercules, CA, USA) to determine fold changes in lnc-CC3, E-cadherin, β-catenin, Snail, and Slug; β-actin was used as an internal control (*n* = 3). Data were analyzed using the 2^−ΔΔCT^ method.

### Rapid amplification of cDNA ends (RACE)

The RNA used to generate cDNA for RACE assays was obtained from SiHa cervical cancer cells. The RACE program was used according to the manufacturer's instructions with the Smarter RACE cDNA Amplification kit 5′/3′ (Clontech, Mountain View, CA, USA). Gene-specific primers for 5′-RACE (GSP1), 3′-RACE (GSP2), and each nest PCR NGSP1 and NGSP2 were designed in accordance with the BE543248 sequence (Table S1). The PCR product was separated on 1% agarose gels containing ethidium bromide. The isolated fragments were cloned into a pMD18-T PCR cloning vector for sequencing (Takara, Dalian, China).

### Northern blotting assay

The DIG Northern Starter Kit (Roche Applied Science, Mannheim, Germany) was used for the northern blotting assay. Primers were designed to amplify the fragment for RNA probe labeling with digoxigenin-11-UTP labeling mix via *in vitro* transcription, and the T7 promoter sequence was added to the 5′-end of the anti-sense primer (Table S1). Five μg per lane of total RNA from SiHa cells was loaded, run on a formaldehyde gel, then transferred onto positively charged nylon membranes using the capillary transfer method. Membranes were fixed at 80°C for 2 h, then prehybridized at 65°C for 1 h in DIG Easy Hyb. Hybridization was performed at 62°C for 16 h in DIG Easy Hyb containing 100 ng/mL DIG-labeled RNA probe. Stringency washes and immunological detection were performed according to the DIG Northern Starter Kit instructions. Hybridization was then visualized via exposure to the imaging device for 2–30 min (Bio-Rad, Philadelphia, USA).

### *In situ* hybridization assay

RNA probes were prepared for northern blot assays using the Roche digoxin probe labeling system. 2 × 10^5^ cells/mL were seeded on a glass slide, incubated in FBS-free 1640 medium for 12 h at 37°C with 5% CO_2_, washed 3 times with 0.1 M PBS (PH7.4), fixed with 4% paraformaldehyde with 0.1% DEPC for 25 min, and then washed with distilled water. 0.5% H_2_O_2_/MeOH was used to remove endogenous peroxidase, followed by a 30 min wash with distilled water. A 3% pepsase solution in citric acid was used to expose RNA fragments, followed by 3 × 5 s washes with 0.5 M PBS. The Enhanced Sensitive ISH Detection Kit I (BOSTER, WuHan, China) was used to perform the hybrid process. Pre-hybridization was followed by hybridization with 50 ng of digoxin-labelled probe; the probe was omitted from negative controls. Saddle brown staining indicated a positive result, and photographs were taken at 200× magnification. The tissue microarray (Auragene, ChangSha, China) *in situ* hybridization assay was conducted using the same protocol after dewaxing.

### Establishment of stable lnc-CC3 overexpression and knockdown SiHa cells

Total RNA was extracted from SiHa cells for first-strand cDNAs synthesis using the First Strand cDNA Synthesis kit (Fermentas, Waltham, MA, USA). Primers for CC3 full length amplification were synthesized with Hind III and BamH I restriction sites (Table S1). The purified PCR product was double digested and ligated into the eukaryotic expression vector pcDNA3.1 (+). The recombinant plasmid was transformed into DH5α *E. coli* cells and confirmed by PCR, double endonuclease digestion, and DNA sequencing. The lnc-CC3 over-expression plasmid was transfected into SiHa cells with Lipofectamine 2000 (Invitrogen, Carlsbad, USA); pcDNA3.1 (+) vector transfected SiHa cells were used as controls. After 48 hours, cells were selected in complete medium containing 800 μg/mL G418 (Sigma Chemicals, St Louis, MO, USA).

Four interfering shRNAs were selected to target lnc-CC3 and expressed using the pSH-U6-GFP (Vigenebio, Rockville, USA) vector. The shRNA sequences were as follows: 1: 5′-CTCTCCTGCTGTCCTCTAATTT-3′, 2: 5′-GGGTCTGCTTTACTTCTAAGGTT-3′, 3: 5′-GCAAG CATCTGCCCTATTTGATT-3′, 4: 5′-GGAAACATGTA AATGGTCTGTTT-3′. Constructed plasmids were transfected into SiHa cells with Lipofectamine 2000 (Invitrogen, Carlsbad, USA); pSH-U6-GFP vector transfected SiHa cells were used as control. Stable clones were selected and grown for one week in complete media containing 8 μg/mL puromycin (Sigma Chemicals, St Louis, MO, USA).

### Cell proliferation test (CCK-8 assay)

In the cell proliferation test, 1000 cells were seeded per well in a 96-well plate with serum-free 1640 medium overnight (*n* = 3). After washes with D-Hanks, cells were incubated in 1640 medium with 5% FBS. Every 24 h for 6 days, 10 μL WST-8 (Vazyme, Nanjing, China) was added to each well followed by 2 h incubation and measurement at 450 nm by spectrophotometer.

### Cell cycle analysis assay

Cells were incubated with serum-free 1640 medium for 24 h, washed with D-Hanks, and cultured in 1640 medium with 10% FBS for 48 h (*n* = 3). Cells were harvested and resuspended in fixation fluid. Cell cycle was detected by FACSCalibur with PI (Becton-Dickinson, New Jersey, USA).

### Colony formation assay

Resuspension fluid with 200 cells was added into 1640 medium with 10% FBS in 6-well plates (*n* = 3). After 14 days, effective colony rates were counted. Colonies containing more than 50 cells were counted as effective colonies. Effective colony rate = (number of colonies counted/numbers of cells seeded) × 100%.

### Wound healing migration assay

Cells were seeded in 6-well plates with 2 × 10^5^ cells per well and grown to 90% confluence. A linear wound was made by scratching the monolayer with a sterile 200 μL |pipette tip, followed by incubation in 1640 medium containing 3% FBS and 3 washes with D-Hanks (*n* = 3). Photographs were taken at 0 h and 48 h after wounding by phase contrast microscopy at the same location in each well (Nikon, Japan).

### Transwell assays

Transwell chambers (Costar, Corning, NY, USA) with 8 μm pore polycarbonate filters were coated with matrigel (BD Biosciences, New Jersey, USA). 2 × 10^4^ cells were seeded into the upper chambers in serum-free medium at a volume of 200 μL per well. The lower chamber contained 500 μL of 1640 medium with 10% FBS as a chemo-attractant. Cells on the upper surface of the chamber were removed after incubating for 48 h at 37°C with 5% CO_2_, fixed with 4% paraformaldehyde, and stained with 0.1% crystal violet solution. Invaded cells in the lower chamber were counted and photographed using phase contrast microscopy. The cell migration assay was conducted similarly but without matrigel. The numbers of invaded cells were determined by photographing in five random views per chamber at 200× magnification.

### Tumor xenografts in nude mice

Five- to six- week old female nude BALB/C-nu mice were purchased from Hunan SJA Laboratory Animal Co. Ltd. 4 × 10^6^ cells suspended in 100 μL PBS were injected into the tail vein. Each group contained 6 mice. After 8 weeks, the mice were sacrificed and the lungs were dissected. The metastatic nodules on the lung surface were counted under stereomicroscope (Olympus, Japan). Lungs were fixed in 4% paraformaldehyde, and paraffin sections were made and stained with hematoxylin and eosin (H&E). Animal experiments followed protocols approved by the Institutional Animal Care and Use Committee of Central South University (Changsha, China).

### Western blotting

Cells were lysed in RIPA buffer containing 1 mM PMSF. Cell lysates were separated by 10% SDS-PAGE, and then transferred onto a NC membrane. The membrane was blocked in PBST with 5% w/v nonfat dry milk at room temperature for 1 h, then incubated with primary antibody (1:1000) in PBST with gentle agitation at 4°C overnight. Primary antibodies were from the Epithelial-Mesenchymal Transition Antibody Sampler Kit (Cell Signaling Technology, Danvers, USA), and β-actin was used as a control (Proteintech, Wuhan, China). After washing three times with PBST, the membrane was incubated with a secondary antibody (1:2000) for 60 min in PBST. The signal was visualized using an ECL detection reagent and imaged at the appropriate time (Bio-Rad, Philadelphia, USA). Densitometry results were quantified using ImageJ software (http://rsb.info.nih.gov/ij).

### Statistical analysis

Student's *t*-test was applied for comparing differences between groups. Overall survival (OS) was calculated using the Kaplan-Meier method. The presence of elevated lnc-CC3 levels in cervical cancer tissues of different stages was evaluated using the χ^2^ test. All statistical analyses were performed using SPSS software, version 19.0 (IBM, Chicago, IL, USA); *p* < 0.05 indicated a statistically significant difference. Figures were made using GraphPad Prism 5 (GraphPad, La Jolla, CA, USA).

## SUPPLEMENTARY FIGURES AND TABLES


