# Supplementary material for: Lnc-CC3 increases metastasis in cervical cancer by increasing Slug expression
Source: Oncotarget. 2016 May 20;7(27):41650–61. doi: 10.18632/oncotarget.9519 (PMC5173085; doi:10.18632/oncotarget.9519)
Supplement: Supplementary file 1 [file oncotarget-07-41650-s001.pdf]

Lnc-CC3 increases metastasis in cervical cancer by increasing Slug expression

Supplementary Materials

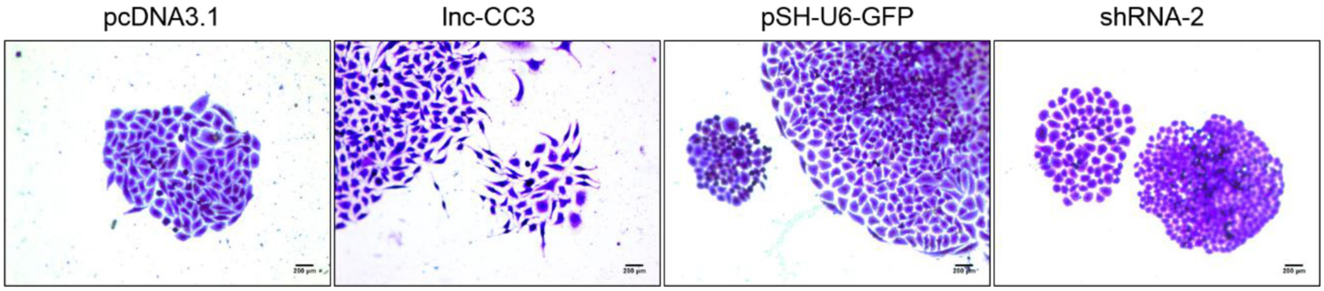

Supplementary Figure S1: The influence of lnc-CC3 in SiHa cell shape structure. The change of lnc-CC3 expression made SiHa colony size obvious difference in colony formation assay. The lnc-CC3 over-expression SiHa cells are longer and expanded growing, while lnc-CC3 knockdown SiHa cells are round and compact growing. photograph at 200× magnification, scale bar = 200 μm. The pcDNA3.1 and pSH-U6-GFP plasmid transfected SiHa cell lines as controls, lnc-CC3 represent over-expressed lnc-CC3 SiHa cell line, shRNA-2 represent lnc-CC3 knockdown SiHa cell line.

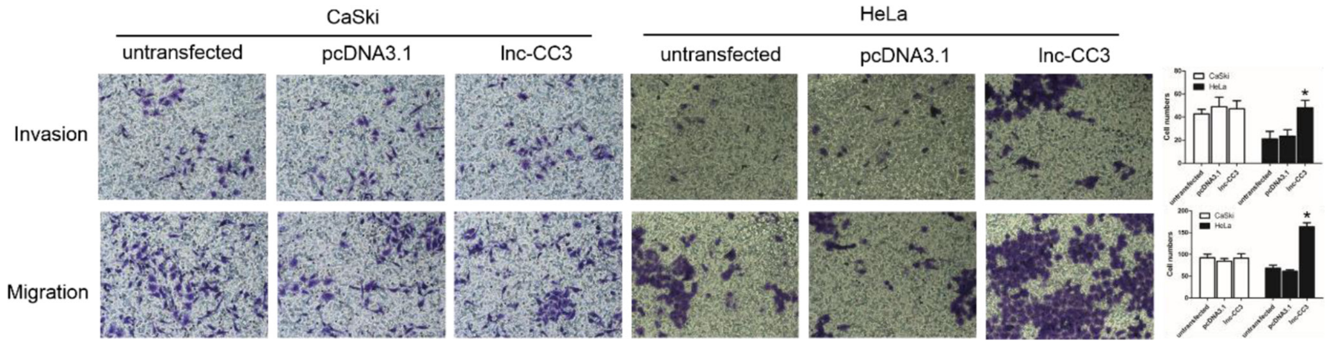

Supplementary Figure S2: The transwell results of lnc-CC3 over-expression CaSki and HeLa cell. Representative images of the migration and invasion transwell assay; invaded cell number was determined by photograph at 200× magnification in five random views per chamber. Untransfected and pcDNA3.1 (+) plasmid transfected cell lines were used as controls; lnc-CC3 indicates lnc-CC3 overexpressing cells. Data are expressed as mean ± SD of independent experiments, *$p < 0.05$.

Supplemental Table S1: Clinical data of tumors with mRNA data(RNA Seq V2)in TCGA database.

See Supplemental Table S1

Supplementary Table S2: Patient clinicopathological characteristics associated with level of lnc-CC3 expression in cervical cancers on tissue microarrays

Sample characteristics	LncRNA CC3 expression, No. of patients			*p*
	Negative	Positive	Total (%positive)	
Age at diagnosis				0.537
< 55 y	38	16	54 (29.6)	
≥ 55 y	8	5	13 (38.4)	
Sample status				0.003
I	17	3	20 (15)	
II	16	4	20 (20)	
III	13	14	27 (51.8)	
Normal	10	0	0	

Statistical significance (*p* value) was computed by χ^2 test.

Supplementary Table S3: The clinical data and in situ hybrization result of tissue microarrays. See Supplementary_Table_S3
